# Determination of Copolymer Block-Length Distributions Using Fragmentation Data Obtained from Tandem Mass Spectrometry

**DOI:** 10.1021/acs.macromol.5c00297

**Published:** 2025-06-16

**Authors:** Tijmen S. Bos, Rick S. van den Hurk, Ynze Mengerink, Ton Brooijmans, Ron A. H. Peters, Arian C. van Asten, Bob W. J. Pirok

**Affiliations:** † Analytical Chemistry Group, Van ‘t Hoff Institute for Molecular Sciences, 1234 University of Amsterdam, Amsterdam, 1098 XH, The Netherlands; ‡ Centre for Analytical Sciences Amsterdam (CASA), Amsterdam 1098 XH, The Netherlands; § Biomedical, DSM, Geleen 6160 BB, The Netherlands; ∥ Brightlands, Geleen 6167 RD, The Netherlands; ⊥ Group Innovation & Sustainability, Testing, Analytics and Physics Group, 63262 Covestro (Netherlands) B.V., Waalwijk 5145 PE, The Netherlands; # Co van Ledden Hulsebosch Center (CLHC), Netherlands Center for Forensic Science and Medicine, Amsterdam 1098 XH, The Netherlands

## Abstract

The block-length distribution (BLD) of polyamide and polyurethane copolymers was determined from mass spectrometry (MS) data. While nuclear magnetic resonance (NMR) traditionally determines the number-average block length, this work demonstrates that MS/MS is a viable option for the characterization of the distribution. An algorithm was implemented and modified to accurately determine copolymer BLDs from fragmentation data. The algorithm incorporates preferences in bond fragmentation. Evaluation of the algorithm encompassed the use of polyamide and polyurethane model systems. In both scenarios, the algorithm successfully derived BLDs, that corresponded well with the average block lengths obtained with ^13^C NMR. The newly developed algorithm enabled the characterization of BLDs based on MS/MS data. Typical trends in BLDs corresponding to distinct synthesis methods were discernible. These BLDs offer uniquely detailed insights into copolymer chemistry, potentially elucidating variations in functional properties of copolymers despite overall identical compositions.

## Graphical abstract

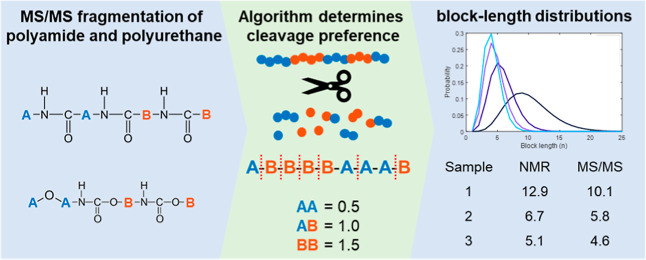

## 1 Introduction

Synthetic polymers are utilized in a large number of products used for different applications such as clothing, cars, solar panels, housing, drug-delivery systems, and biodegradable implants.^1–5^ For most applications, copolymers are used that comprise of multiple different repeating units, also referred to as monomers. The potential application of copolymers seems to be unlimited when it comes to designing tailor-made physical, mechanical, and structural properties. The properties of a copolymer depend heavily on the sequence, type, and number of different monomers as they determine the intramolecular interactions. In general, copolymers are heterogeneous and their chemical (or molecular) structure cannot be described by singular values, but rather by distributions.^6^ These chemical distributions include but are not limited to, the molecular-weight distribution (MWD), chemical-composition distribution (CCD), branching distribution (BD), functionality-type distribution (FTD), branching distribution (BD), and block-length distribution (BLD).^1,7^ The molecular structure encompassing these distributions directly translates into its macroscopic properties.^8^ To establish structure–property relationships it is essential to accurately characterize the molecular structure of copolymers, including the BLD.

The BLD is of high interest to the design of advanced materials with specific properties. Block structures within copolymers can be used to create and control so-called microphase-separated structures.^9,10^ For instance, block copolymers can be produced with a constant average chemical composition (e.g., 50% monomer A and 50% monomer B) but with different average block lengths and BLDs. Noro et al. demonstrated that for a relatively simple diblock and triblock structure, the microdomain spacing increased with increasing BLD width (dispersity of a block type) at constant chemical composition.^9^ They furthermore demonstrated that triblock copolymers arrange into regular microphase-separated structures more easily than diblock copolymers which directly impacted the macroscopic properties. The role of the BLD in the formation of solid-state structures has also been investigated for polypeptide-styrene copolymers.^11^ The resulting morphology was a plane lamellar structure that was disrupted according to the length of the helices, indicating the influence of the BLD on the interface curvature properties. Another example of macroscopic properties determined by the block structure is in self-assembling methacrylic acid (MAA) and methyl methacrylate (MMA) copolymers. The shape of the copolymer in solution was dependent on the polyMAA and polyMMA block lengths.^8^ Block copolymers are also used for nanoparticle-based drug-delivery systems where the BLDs influence the radius of the self-assembling micelles in solution.^12^ In the case of thermoplastic polyurethanes (TPU), these microphases are built up from the soft and hard blocks in the polymeric chains. The hard segments may create locally crystalline structures by coordinating with other hard blocks of either the same or different polymer chains, thereby contributing to the strength, rigidity, and melting point of the material. The soft segments are typically polymeric diols which give a flexible amorphous structure that influences the elasticity and flexibility and selective migration of small molecules through the soft domains. The average and dispersity of these BLDs impact the thermal and mechanical properties of TPU.^13–18^ For example, a higher Young’s modulus with increasing hard-block lengths has been demonstrated as well as differences in water vapor permeability.^19,20^ With regard to the dispersity of the distribution, it has been demonstrated that narrow distributions exhibit a higher modulus and thermal resistance.^18,21,22^

To acquire information on the block length of copolymers, the polymer must be reduced to smaller, detectable fragments. This can be achieved through sample transformation.^6^ For synthetic polymers, this can be accomplished by using pyrolysis gas chromatography (py-GC) coupled with mass spectrometry (MS).^23,24^ By performing pyrolysis, small oligomers are obtained (mostly monomers, dimers, and trimers). Through quantification of the trimers, the blockiness of the polymer can be estimated. The fragmentation process in pyrolysis is random and models have been developed to correct for this effect.^25^ However, the length of detectable fragments using py-GC-MS is limited, and longer fragments provide more detailed information about the sequence than shorter ones. Consequently, only the average block length can be determined. More recently, MS-based fragmentation has also been explored to elucidate the sequence of copolymers. This approach has predominantly been applied to copolymers with low molar masses (typically <5000 Da), using liquid chromatography (LC) coupled to tandem MS (MS/MS) or matrix-assisted laser desorption ionization (MALDI)-MS/MS.^26–29^ In such cases, the experimental design aims to limit the number of different molecular species introduced into the mass spectrometer simultaneously, thereby producing clean, interpretable spectra. Additionally, the use of low molecular weight species allows for the isolation of singly charged precursor ions, enabling selective fragmentation and analysis at the tandem mass spectrometry level. However, for copolymers with higher molar masses (number-average molar mass > 20,000 Da), the detection of singly charged ions becomes impractical. As a result, the full envelope of multiply charged ions is typically subjected to fragmentation in a nonselective manner, as demonstrated by Mengerink et al.^30^ The authors, demonstrated MS-based fragmentation of polyamide copolymers and detecting fragments up to the length of 10 repeating units.^30^ For the first time, the BLD could be determined from these fragments using a Monte Carlo (MC)-based algorithm. Algorithms that can transform quantitative copolymer fragment data into BLDs are referred to as SWAMP (Systematic workflow for analyzing multifragmented polymers). To verify these fragmentation methods and develop functional models, reference data was obtained from ^13^C nuclear magnetic resonance (NMR). In previous work, we recently introduced a machine learning-based algorithm that can more accurately reconstruct the block-length distribution from fragment data.^31^ Rather than analytical reference data, simulated ground-truth fragment data was used to develop, optimize, and efficiently test this algorithm to perform SWAMP. However, an actual polymer sample cannot be characterized in silico, some form of quantitative instrumental data is required to apply the new algorithm in a meaningful manner in practice.

This study focuses on elucidating the BLDs of copolymers using MS-based fragmentation data, that is interpreted by a machine learning-based algorithm to derive accurate distributions. The proposed SWAMP workflow was validated using experimental data of two types of copolymers: polyamides and polyurethanes. While polyamide contains polyamide-4,6 and polyamide-4,10, polyurethane is made from (poly)-diols and diisocyanate. The polyamide copolymer was utilized to validate the capability of processing quantitative data into the expected BLDs. The polyurethane copolymers were expected to have a larger difference in fragmentation and ionization. The previously published analytical solution was extended to incorporate cleavage preferences. To assess the ability of the proposed methodology to arrive at meaningful BLDs, five polyurethanes were synthesized, all sharing the same overall chemical composition and comparable molecular weight but with varying block length distributions due to different synthesis routes. The polyamide fragment data was obtained from earlier published work from Mengerink et al.^30^ These polyamide samples have the same overall composition but differ due to transamidation in time. The number-average block lengths (NABL) and overall chemical composition of these products were determined with NMR spectroscopy. To prevent interference from small oligomers when introducing samples into the MS, LC (liquid chromatography)-MS/MS was employed.

## 2 Materials and Methods

### 2.1 Chemicals

The water used in this work was deionized (Arium 611UV; Sartorius, Germany; resistivity 18.2 MΩ cm). Unstabilized tetrahydrofuran (THF, HPLC grade) and *N*,*N*-dimethylformamide (DMF) were obtained from Biosolve (Valkenswaard, The Netherlands). Formic acid (FA, ≥98% (v/v)) was obtained from Merck (Darmstadt, Germany).

1,4-Butanediol (BDO) was obtained from BASF (Ludwigshafen am Rhein, Germany). Stannous octoate was obtained from Sigma-Aldrich (Darmstadt, Germany). Poly tetrahydrofuran (pTHF, *M*_n_ = 1000 Da) was obtained from InnoSyn (Geleen, The Netherlands). Methylene diphenyl diisocyanate (MDI) was used as provided by Thermo Scientific Chemicals (Geel, Belgium). Tetrachloroethane-D2 (C_2_D_2_Cl_4_), and DMSO–D6 were obtained from Sigma-Aldrich (Darmstadt, Germany). For chemical and synthesis information on the polyamides see Mengerink et al.^30^

### 2.2 Procedures

#### 2.2.1 Synthesis of Model Polymers

In this research, the TPUs are synthesized using three monomers, a polyether diol, a diisocyanate, and a small diol as a chain extender in the presence of a catalyst (Figure 1). In a two-step reaction, the first step combines the polyol and diisocyanate to form an alternating structure that is NCO-terminated resulting from excess isocyanate. In the second step, the chain extender is added, and in some cases additional diisocyanate, to connect the soft blocks with alternating diisocyanate and chain extender. Using the same chemical composition, different block lengths may be achieved by controlling the NCO/OH ratio in the first reaction step and by adding the remaining diisocyanate in the second step.

**Figure 1:**
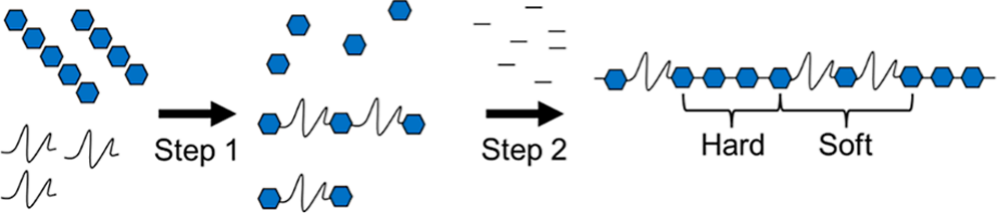
Generic reaction scheme of the two-step polyurethane synthesis where the blue hexagons represent the diisocyanate, long curved lines indicate the polyether-diol, and short straight lines illustrate small diol chain extenders. In step one, an NCO/OH ratio of 3 is depicted and no additional NCO is added in step two. The hard and soft blocks are highlighted in the final product.

Model TPUs with a deliberate variation in the distribution of block lengths were synthesized for research purposes. For the synthesis of the model compounds, solutions of reactants in anhydrous DMF were first prepared and reacted in one or two steps according to the scheme in Table 1. All samples were prepared using a formulation consisting of 45.0 wt % MDI, 42.8 wt % pTHF, and 12.2 wt % BDO, corresponding to approximate molar fractions of 50% MDI, 12% pTHF, and 38% BDO. The composition of the samples determined with NMR is shown in Table 1. Solutions of reactants were prepared in anhydrous DMF. For the one-shot sample, a solution containing the MDI was added in one step (5 s) to a solution containing all other reactants and stirred at room temperature (RT) for 16 h. For the two-shot samples, in the first step, a solution containing a fraction of the MDI was added (5 s) to a solution containing the pTHF and stirred at RT for 6 h. In the second step, a solution containing the BDO and a solution containing the remainder of the MDI was added to the mixture and stirred for 16 h at RT. The resulting mixtures were precipitated by the addition of water until turbid followed by slow stirring for 1 h. The resulting product was washed using water, methanol, and water and dried for 16 h at 40 °C at 400 mbar. To remove residual DMF, the material was cut in pieces and stirred in water at RT for 24 h after which it was dried at 40 °C at 100 mbar. This last step was repeated two more times.

**Table 1:** Mol Ratios Used for the Synthesis of the Five Polyurethane Samples with Varying Block Lengths and Composition Determined with NMR^a^

sample name	NCO/OH in the first reaction step	weight fraction pTHF (%)	weight fraction BDO (%)	weight fraction MDI (%)	*M*_n_	*M*_w_
one-shot (OS1)	not applicable	43.6	11.2	45.2	31,500	56,300
two-shot 1 (TS1)	1.1	44.7	9.9	45.5	18,000	39,000
two-shot 2 (TS2)	2	43.6	10.6	45.8	30,100	48,900
two-shot 3 (TS3)	3	43.8	10.8	45.4	23,300	40,600
two-shot 4 (TS4)	4.4	45.1	10.0	44.9	25,800	39,600

a Molecular weights were determined with SEC.

The one-shot (OS) sample represents a statistical distribution as all components are mixed in one step. The two-shot (TS) series is designed to systematically increase the weight-average size of the hard and soft segments by splitting the addition of MDI between the first and second steps. For example, TS1 has a 1.1:1 molar ratio of MDI to pTHF, leading to long soft block prepolymer (MDI-[pTHF-MDI]_*n*_) which would result in large block lengths of the hard and soft blocks in the final product. In TS4, all MDI is added in the first step resulting in lower expected block lengths. TS2 and TS3 represent intermediate products. In all cases, the total NCO/OH ratio of the starting materials was maintained at 1:1. As a result, the second step inherently retains a 1:1 NCO/OH ratio, irrespective of the specific components used in the first step. This outcome arises because any NCO excess from the first step carries over and remains reactive in the second step.

#### 2.2.2 NMR Measurements

The NMR instrument used in this study was a Bruker Avance III 500 MHz spectrometer (Bruker Biospin GmbH, Germany) equipped with a 5 mm cryogenically cooled probe head. The sample solutions were prepared by dissolving approximately 15 mg sample in 600 μL tetrachloroethane-D2 (C_2_D_2_Cl_4_) at 100 °C for ^13^C NMR or by dissolving in DMSO–D6 at 45 °C for ^1^H NMR. The carbon-13 NMR spectra were processed using a line-broadening factor of 0.1 to enhance the signal-to-noise ratio while minimizing distortion of the peak shapes. The blockiness of the polymer sample was determined with ^13^C-wheare as the monomer composition was established with ^1^H NMR.

The NABLs were determined from the ^13^C NMR spectra using the equations reported by de Ilarduya et al.^32^ The spectra were deconvoluted by fitting four Lorentz distributions to the relevant aromatic regions (136.349, 136.363, 136.467, and 136.488 ppm) using a Nonlinear least-squares solver through the use of the Matlab function “*lsqnonlin*”.

#### 2.2.3 LC–MS/MS Measurements

The LC–MS/MS experiments were performed using an Agilent 1290 Infinity II system comprising of a quaternary pump (G7104A), sampler (G7129B), column oven (G7130A), and a diode-array detector (DAD) (G7117A) equipped with an Agilent Max-Light cartridge cell (G4212-60008, 10 mm, *V*_*det*_ = 1.0 μL). This LC system was coupled to an Agilent Q-TOF (G6550A) mass spectrometer to perform the MS/MS measurements. The flow from the LC was first directed to the DAD which was then directly coupled to the ESI (electrospray ionization) source of the MS instrument.

A Waters Acuity UPLC BEH C18 (100 × 2.1 mm, 1.7 μm) column was used and operated at a column temperature of 50 °C. Mobile phase A consisted of water with 0.1% (v/v) FA and mobile phase B consisted of unstabilized THF with 0.1% (v/v) FA. The flow rate was set to 0.2 mL min^–1^ starting at 20% B. A linear gradient to 95% B was performed from 0 to 35 min. It was then held at 95% B for 5 min after which it returned to 20% B in 1 min as starting condition. UV absorption was monitored at 254 nm using a bandwidth of 4 nm at a frequency of 5 Hz. The ESI source was operated in positive ion mode and collision-induced dissociation (CID) was performed with varying voltages (0–40 V) for fragmentation of the copolymers. No ion selection was applied in the first quadrupole. All incoming ions were subjected to CID, and the resulting MS^2^ spectra were used for interpretation. For a detailed overview of the instrumental settings of the MS, see Supporting Information Section S-1. All MS spectra were recorded in profile mode. The samples were prepared by dissolving the polymer samples in DMF at a concentration of 2 mg mL^–1^, subsequently, 1 μL of the polymer solution was injected into the LC–MS/MS system. For methods used for the analysis of the polyamides see Mengerink et al.^30^

#### 2.2.4 Data Processing

All code was written in-house using Matlab 2024a (Mathworks, Natick, MA, USA).

##### 2.2.4.1 LC–MS/MS Data Extraction

The LC–MS/MS data of the TPU samples was extracted by taking the sum of all fragmentation spectra across the elution peak of the polymer. For example, all spectra from 29 to 33 min are binned and summed to obtain one average MS spectrum containing the raw fragment data. The bin size used is dynamic, changing as a function of *m*/*z*, as default from MassHunter (Agilent, Waldbronn, Germany) (0.0003*m/*z* ^0.5^). To obtain the relevant peak intensities, the *mspeaks* Matlab function is used to obtain a centroid peak list. The *m*/*z* axis was corrected through internal calibration based on the protonated MDI-BDO fragment. The entries of the centroid peak list were matched to the closed matching *m*/*z* value of interest within 0.01 *m*/*z*. For this purpose, repeating unit A was defined as pTHF and B was MDI-BDO (see Figure 2), and observed fragments were protonated resulting in eq 1 to compute the expected *m*/*z* of any given A_*n*_B_*m*_ fragment. Only singly charged protonated combinations of the targeted monomers were selected from the mass spectra, based on the *m*/*z* values defined in eq 1. This strategy facilitates broader applicability to various polymers, as only the repeating units need to be specified, without requiring complete knowledge of all possible fragmentation pathways. When enough relevant fragments are detected and fragmentation preferences are considered, this approach enables robust estimation of block-length distributions. The signals of interest, while still well detectable, accounted for approximately 2.7% of the maximum signal intensity. The intensities of each extracted mass were adjusted using the theoretical isotope composition to enhance signal quantification. Between 44 and 48 signals of interest were identified in the MS^2^ spectra. For computational purposes, only fragments up to five repeating units were used, as this approach has been shown in previous work to be sufficient while ensuring feasible computation times. One consideration when utilizing MS^2^ spectrometry data is the potential overlap between relevant fragment signals and unrelated peaks. In this study, such overlap appeared to have minimal impact, as verified by careful peak inspection. An example spectrum and annotated peaks of interest are provided in Supporting Information Section S-2 Figure S-1 and S-2.

1 Monoisotopicmass=n×72.057515+m×340.142308+1.007825

**Figure 2:**
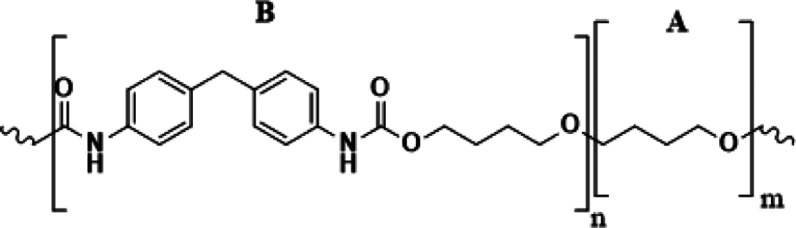
Definition of the two segments extracted from the MS/MS data. A corresponds to pTHF and B to MDI-BDO repeating units.

##### 2.2.4.2 Generation of Fragment Tables

The previously published algorithm to generate fragment tables from BLDs systematically evaluates each index of the sequence by iterating through combinations of A and B repeating units, forming the base segments.^31^ The algorithm computes the count of A and B repeating units within the fragment length at each index of the base segment. If the sum of the desired fragment length and the current sequence length, initiated with the base segments, exceeds the total sequence length, all unique block combinations are appended. This process continues until the sequence surpasses the required length. Once the appropriate length is achieved, the contribution of all possible combinations is computed. Subsequently, the algorithm calculates the count of A and B at each index for the desired fragment length, multiplying them by their respective abundances as determined by the BLDs. Additionally, it assigns weights to these counts based on the proportion of monomers included in the initial A and B blocks. The machine learning (ML) algorithm utilizes this algorithm to evaluate which BLDs result in specific fragment tables. It learns from this information to determine the underlying BLDs. The reader is referred to our previous article for further information on the computations.^31^ To apply the algorithm to MS/MS data, preferences in cleavage based on bond type, ionization efficiencies, charge localization, and other effects have been incorporated. The algorithm describes such preferences as a ratio between available bond types. For typical copolymers, encompassing AA, AB, and BB bonds, an illustrative ratio could be represented as [0.5, 1, 1.5], an example shown in Figure 3. See Figure 4 for a flowchart of the complete workflow.

**Figure 3:**
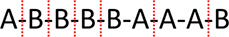
Example of cleavage preference of AA = 0.5, AB = 1, and BB = 1.5.

**Figure 4:**
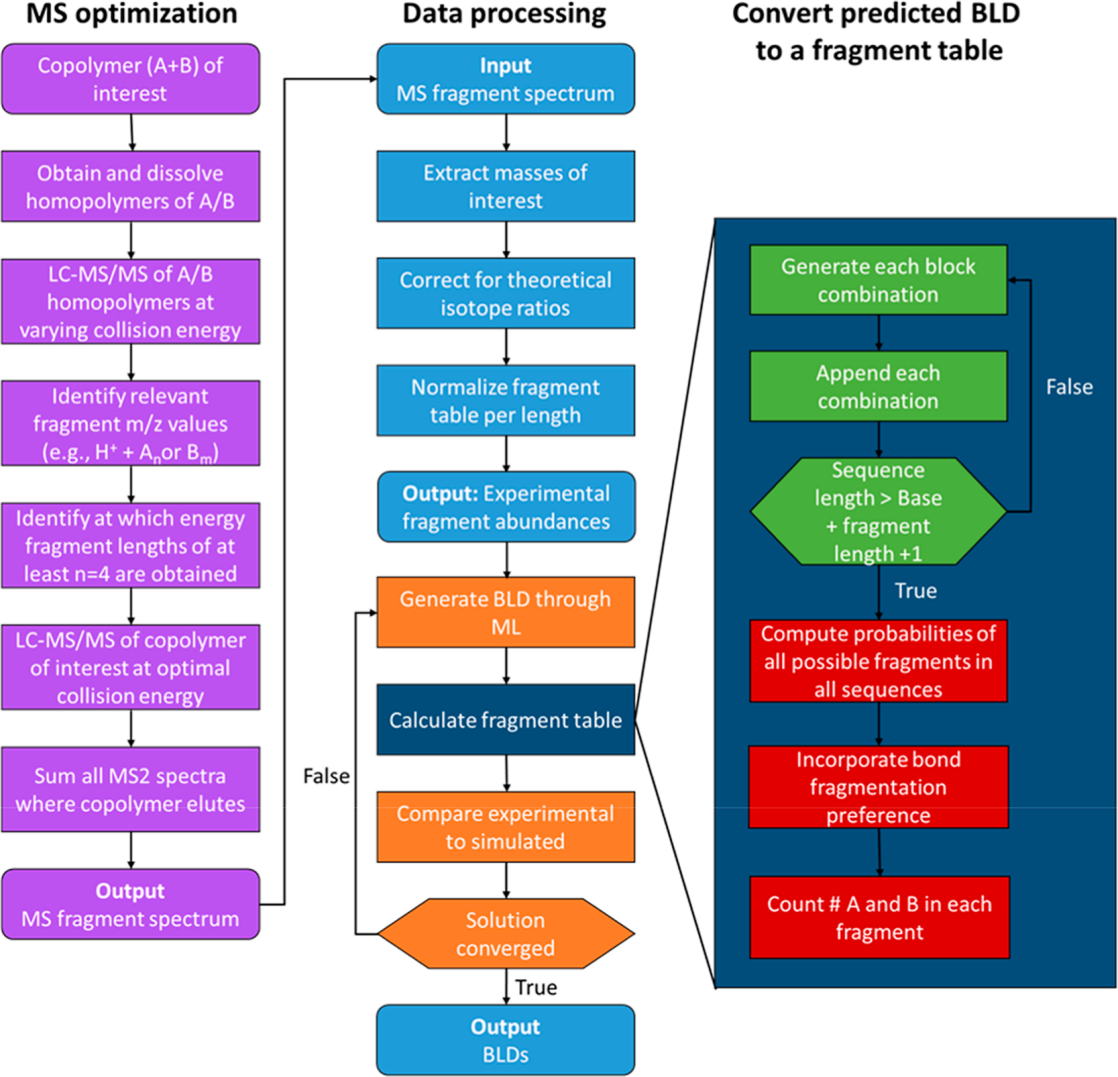
Flowchart illustrating the proposed SWAMP workflow for determining BLDs from a fragment table obtained via MS/MS. Light blue represents data input, output, and preprocessing. Purple indicates parts of the MS optimization and orange indicates parts of the ML algorithm. Dark blue represents the algorithm that calculates the fragment table originating for BLDs. Within this algorithm, green represents the sequence generation, and red the computation of the abundances.

In this study, the Schulz-Zimm distribution was employed, chosen for its versatility in accommodating a broad spectrum of copolymers and its ability to transition between Flory and Gaussian distributions.^33,34^ The code of the algorithm for generic copolymers is provided at github.com/TSBOSuva/BlockLengthDistributionModel and in Supporting Information—Analytical solution workflow code.

## 3 Results and Discussion

### 3.1 Block Length Determination on Poly Amide Copolymers

In the present study, we used a relatively straightforward case involving a polyamide-4,6/polyamide-4,10 copolymer of which quantitative data was available to validate the new algorithm. This copolymer features only one specific bond between its monomers, as highlighted in red in Figure 5. While other bonds, particularly internal amide bonds, can be cleaved, only fragments containing complete monomers are considered in the analysis.

**Figure 5:**
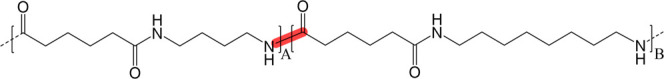
Structure of polyamide-4,6/polyamide-4,10 copolymer. Red indicates the bond of interest.

Using this polymer model as a stepping stone, we could establish the performance of the ML when realistic noise on the experimental fragment data was exclusively incorporated as no fragmentation preference had to be included in the model yet. As our ML workflow has thus far only been applied under the assumption that fragmentation occurs randomly data corrected for fragmentation preferences was required. The availability of both polyamide polymers as homopolymers facilitated the calibration of the fragment abundance data, thus providing quantitative data. Mengerink et al. generated this MS/MS data set as well as the, which were used to demonstrate their MC-based SWAMP approach for determining BLDs in copolymerized polyamides synthesized under varying extrusion conditions.^30^ The MC-based algorithm with the α-Ω rule devised by the authors is particularly suitable for Flory-like distributions, which aligns with the characteristics of the investigated polyamide. Their study involved comparing the averaged BLDs across both polyamide-4,6 and polyamide-4,10, revealing a consistent trend, see Figure 6. The ML-based algorithm was applied to this existing data set for the purpose of comparison. The BLDs obtained using the ML-based algorithm indicate that polyamides with longer reaction times exhibit lower and narrower distributions, while those with shorter reaction times display higher and broader distributions compared to those obtained the previous MC-based algorithm, as illustrated in Figure 6A,B. A similar effect was observed in our previous work where the new algorithm was compared to the MC-based algorithm on synthetic data sets where the MC-based algorithm tended to overestimate the block lengths.^31^ This effect was more pronounced for shorter block lengths due to the limitations of the MC-based algorithm. Looking at the individual BLDs generated by our new algorithm, polyamide-4,6 exhibited a slightly higher NABL than the NABL of polyamide-4,10, see Table 2. This is particularly evident in samples subjected to shorter reaction times. This difference was related to the chemical composition of the copolymer which was consistent with data from the original paper. The Composition obtained through the ML algorithm matches well with the composition determined with NMR, see Table 2.

**Figure 6:**
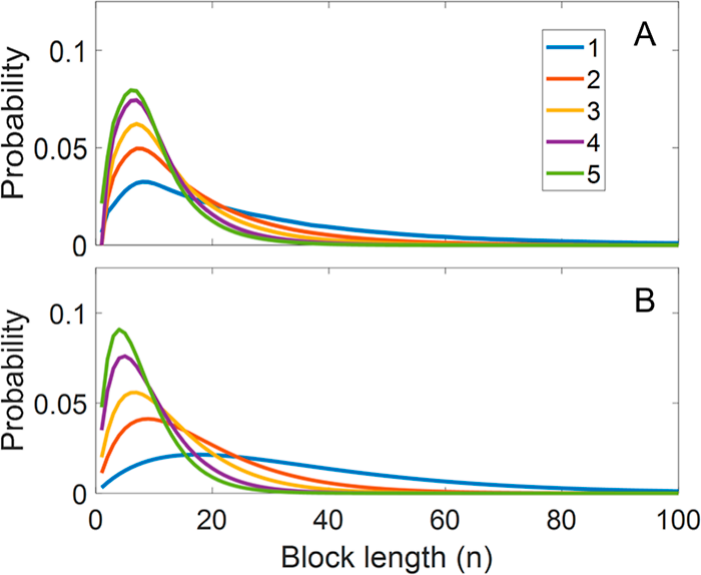
BLDs of 5 polyamides. The numbers 1 through 5 refer to the reaction time in minutes, where longer reaction times result in more transamidation and therefore shorter expected block lengths. The probability corresponds to the number fraction containing the specified block length. Panel A shows the average BLD determined by the MC-based algorithm. Panel B shows the average BLD determined by the ML-based algorithm.

**Table 2:** Overview of Number-Average Block Length (NABL) and Composition of the Analyzed Polyamides^a^

	NABL			composition			
sample	average (MC)	PA-4,6 (ML)	PA-4,10 (ML)	PA-4,6 (NMR)	PA-4,10 (NMR)	PA-4,6 (ML)	PA-4,10 (ML)
T1	26.3	29.4	25.5	57	43	53	47
T2	17.2	16.9	13.4	56	44	56	44
T3	13.6	13.0	9.8	57	43	57	43
T4	11.1	9.6	7.3	56	44	57	43
T5	9.9	7.9	6.2	56	44	56	44

a The NABL data from MC and the NMR composition data was obtained from previously published work.^30^

### 3.2 Block-Length Determination on Polyurethane Polymers

#### 3.2.1 Measuring Polyurethane Fragments with LC–MS/MS

Polymeric products can often contain low molecular weight oligomers or cyclic oligomers as impurities arising from the production process. To mitigate interference from these side products and to enable the study of the BLD of the high-molecular-weight polymer, a conventional RPLC method was employed for the separation of these low molecular-weight components from the copolymeric product. Additional details on the separation of oligomers and the copolymeric product can be found in the Supporting Information, Section S-3.

To facilitate MS/MS fragmentation of the polyurethane, it was imperative to ensure that the polymer acquired sufficient charges during the ionization process, ensuring that enough of the resulting fragments remained charged post-fragmentation. To achieve this, formic acid was added to the mobile phase. We assume that when large copolymers have a similar composition, there are no significant differences in ionization, and only fragmentation effects need to be considered. Evaluation of various CID voltages was undertaken to optimize the sensitivity of the generated fragments. It was crucial to ensure sufficient fragmentation to obtain fragments within the observable *m*/*z* range. However, the CID voltage should not be too high as this would predominantly result in monomer generation rather than fragments of more substantial lengths. Since our main interest was the MDI-BDO distribution, ideal conditions should favor the formation of these fragments. To investigate the effect of CID voltage on polymer fragmentation, model polymers were used. Particularly, a pure pTHF sample was used to model the ether-bond fragmentation and an MDI-BDO alternating sample was used to study the urethane bonds. Figure 7 shows a selection of fragment ions resulting from the fragmentation of pTHF (*n* = 10) and an MDI-BDO (*n* = 6) species while varying the CID voltage from 0 to 40 V. These were chosen as computing fragments longer than 10 monomers is too computationally expensive and for MDI-BDO no fragments longer than 6 monomers were detected. For each bond type, the original and multiple-charged species along with the monomer, and dimer fragments were investigated. In both cases, at 0 V there was some 2+ charge state visible which decreased with increasing CID voltage. Correspondingly, the monomer fragment abundance increased with voltage in both cases. The formation (and later fragmentation) of the dimer-fragment abundance was observed with a tipping point at 25 V for MDI-BDO and around 20 V for pTHF. After this tipping point of pTHF, the most abundant species shifted toward the monomers, which was not ideal for obtaining larger fragments. As shown in our previous work, it is important to have fragments containing at least 4 monomers for an accurate elucidation of the BLDs.^31^ Since the detection of fragments containing MDI-BDO was the most challenging the voltage of 25 V was selected as this voltage showed the most abundance. It is important to recognize that this critical parameter should be evaluated for each polymer type and instrumentation setup individually. Direct comparison across different instruments and polymer systems is likely to be challenging due to inherent variability. While the ionization and fragmentation behavior of these oligomers cannot necessarily be extrapolated to the high molecular weight copolymers, a balance between sufficient fragmentation and not fully fragmenting to monomeric species for the copolymer samples was achieved (Figure S-2).

**Figure 7:**
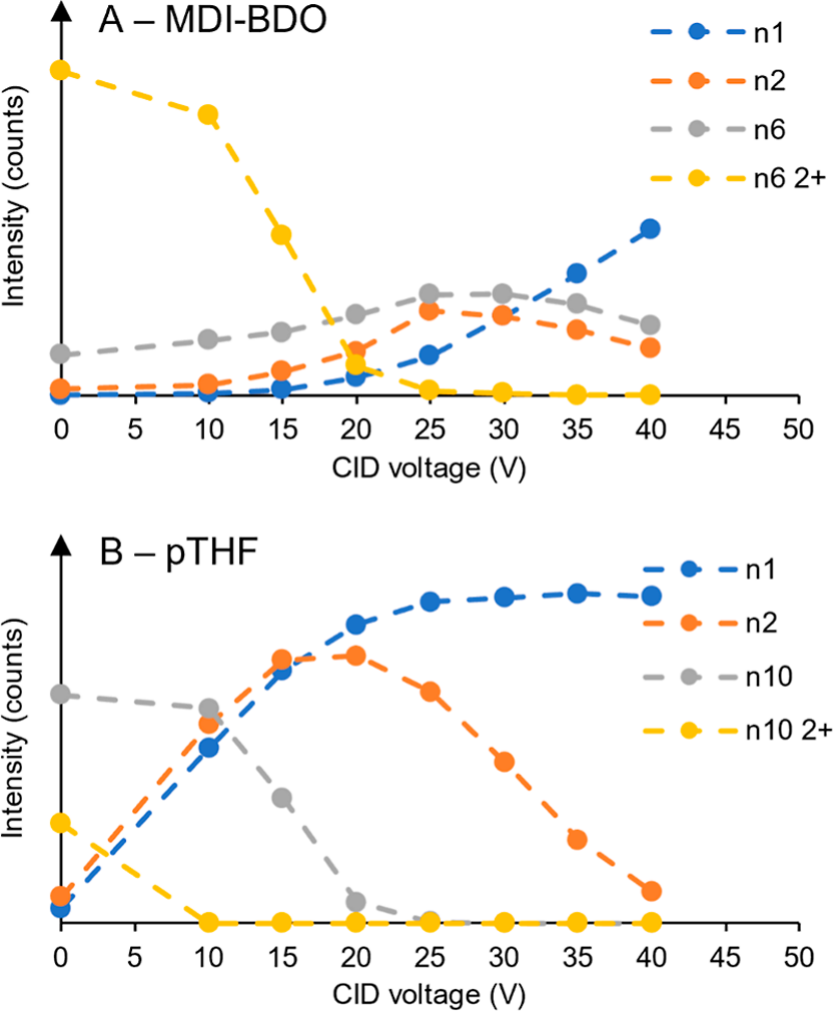
Fragment-ion intensity at varying CID voltages for a selection of fragments of pTHF and MDI-BDO representing the ether and urethane bond types, respectively. Data was obtained by LC–MS/MS, for MDI-BDO (*n* = 6) (A) and pTHF (*n* = 10) (B).

#### 3.2.2 Defining the Chemistry and Fragmentation Pathways

Unlike the polyamides, the polyurethanes investigated here contain two types of cleavable bonds between monomers. This difference introduces potential large variability in bond cleavage, influenced by factors such as bond strength and charge localization. These combined effects can be approximated by a ratio indicating the average effects of fragmentation preference, charge localization, etc. To integrate this concept, the algorithm was adapted based on an earlier-published version that evaluates the known bond type of each fragment. In the context of PU, the BLDs of particular interest are those of the hard block (MDI-BDO). In our specific case, the B repeating unit corresponds to MDI-BDO. Notably, MDI-BDO is considered the repeating unit but contains a urethane bond between the MDI and BDO. Therefore, it is capable of internal cleavage. Thus, a MDI monomer is consistently positioned between two A (THF/BDO) repeating units, allowing only two bond types (urethane and ether) to form. For example, a sequence of BBB could also have fragmented in one of the B repeating units on the end resulting in a detected ABB fragment. While this effect is specific to the chemistry of this copolymer and infrequently encountered, it could be easily incorporated into the algorithm.

Cleavage preference between ether and urethane was incorporated into the machine learning algorithm as an additional parameter, expressed as a ratio. Consequently, at least two samples with identical chemistry but the same or known compositions with different BLDs were necessary for the algorithm to ascertain the fragmentation ratio. In scenarios involving more conventional copolymers containing AA, AB, and BB bonds, the ratio would comprise three numbers, potentially necessitating an additional sample for determination. However, if this ratio has been established previously for the applied setup, a single reference sample could suffice for calibration to correct for day-to-day variation.

Many copolymer BLDs adhere to either a Flory or a normal distribution. In order to enhance the workflow’s applicability across diverse synthesis methods, the Schulz-Zimm distribution, as described in eq 2, was incorporated.^33,34^ This distribution exhibits a spectrum ranging from the Flory to a normal distribution, thereby encompassing a wide array of BLD shapes. In eq 2 *k* represents the polydispersity parameter, *n̅* is the NABL, *n* is the block-length being sampled, while Γ­(*k*) corresponds to the Gamma function of *k*. When *k* = 1 then the function equals an exponential Flory distribution, at large *k* values it approaches a Gaussian, and at *k* = ∞ the distribution becomes infinitely narrow corresponding to a monodisperse multiblock copolymer.

2 $$BLD\left(n\right)=\frac{k^{k}\times e^{-\frac{kn}{\mathrm{n̅}}}\times n^{k-1}}{\Gamma \left(k\right)\times {\mathrm{n̅}}^{k}}$$

In the case of the polyurethanes, the raw data contains additional species with a block length of 1. For the MDI-THF, these single MDI originate from the soft block, whereas for the pTHF distribution, they originate from the BDO in the hard block. However, due to the way repeating unit B is described with the capability to cleave internally, these additional species of length 1 are not visible in the BLDs.

#### 3.2.3 Block-Length Distributions of Polyurethanes

The definition of the polyurethane copolymer allows the algorithm to ascertain the BLDs of interest. Five polyurethane samples were selected, each expected to exhibit distinct hard-block and soft-block distributions while maintaining identical pTHF distributions, as sourced from the same chemical batch. Each sample exhibits a distinct BLD for the hard block segment MDI-BDO, as shown in Figure 8. Figure 8A displays for the OS sample both the pTHF and MDI-BDO BLD, whereas Figure 8B exclusively displays the BLD of MDI-BDO in the hard block of the TS sample series and the pTHF distribution defined by the algorithm, omitting the representation of MDI-THF in the soft block. Notably, the TS samples demonstrate a decreasing trend in the NABL with an increasing NCO/OH ratio in the initial reaction step, a pattern consistently reflected in the determined BLDs. Specifically, the OS sample, synthesized in a single step and thus more random, exhibits a NABL of 5.0, a value within the NABL range for TS2 and TS3.

**Figure 8:**
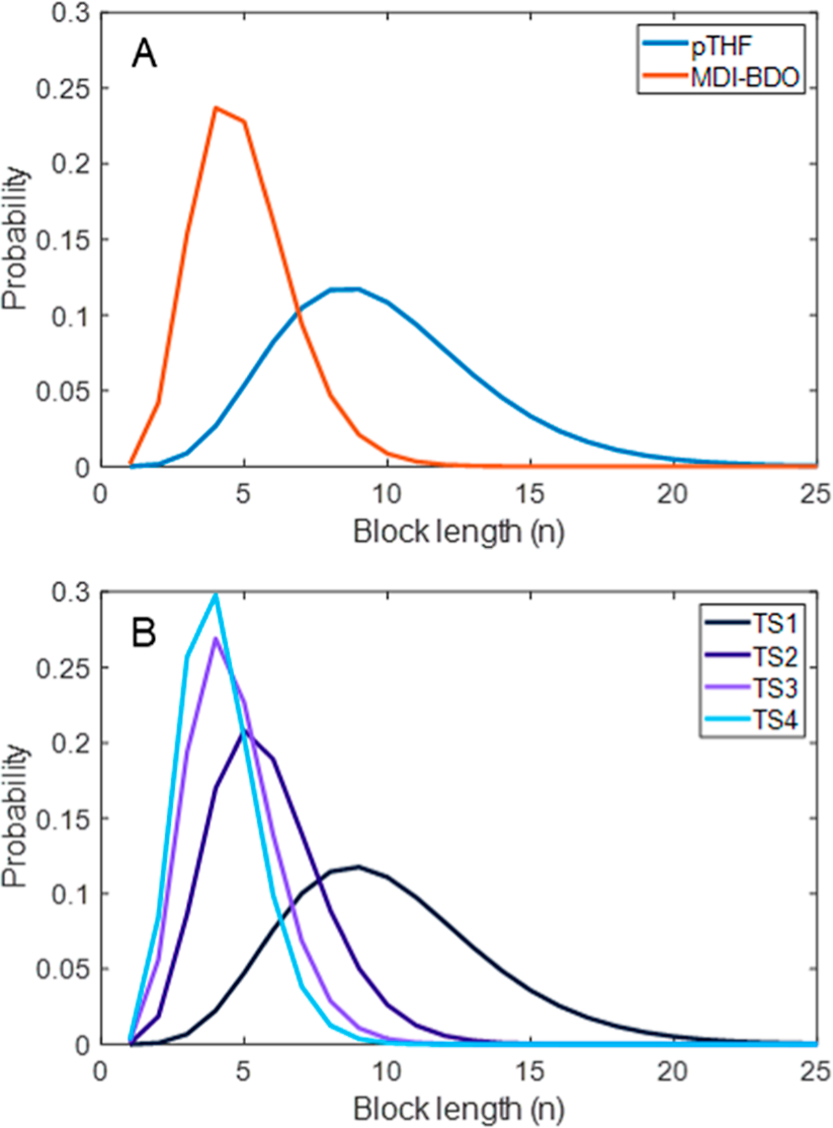
Number-based BLDs of pTHF and hard-block MDI-BDO of the OS sample (A) and the MDI-BDO distributions of the TS samples (B).

In the absence of a standard with a known BLD, an alternative method to assess the quality of BLD determination involves comparing the NABLs obtained from nuclear magnetic resonance (NMR) spectroscopy with those derived from the ML algorithm based on tandem mass spectrometry (MS/MS) results. The NABL of the hard and soft block segment was determined through NMR spectroscopy, however the pTHF segment according manufacturer specifications indicates a number-average molecular weight of 1000 Da, corresponding to a NABL of 13.63 while the NMR shows 9.9 ± 3.3.

The NMR analysis was conducted according to the procedure outlined in the experimental section including deconvolution of the NMR signal. While this method offers a reliable estimation of the hard block lengths, some errors may persist in the integration of the NMR signal, which is especially true for TS1 which shows significant convolution. This is a result of the significant overlap in the ^13^C NMR spectra as can be seen in the Supporting Information, Section S-4. Therefore, the close agreement in relative trend between the NABLs derived from NMR and ML provides confidence that the ML, as indicated in Table 3, workflow produces meaningful BLDs. The 1H-NMR measurements used for the determination of the compositions is reported in Supporting Information Section S-5.

**Table 3:** Composition and NABL of MDI-BDO and pTHF Determined Through NMR and the ML-Based Workflow^a^

	NMR		ML		
sample	composition MDI-BDO/pTHF (wt %)	NABL	composition MDI-BDO/pTHF (wt %)	NABL	*D̵*_BL_
OS1	56.4/43.6	5.4	52.6/47.4	5.0	1.12
TS1	55.3/44.7	16.2*	5*2*.6/47.4	10.1	1.13
TS2	56.4/43.6	5.5	52.6/47.4	5.8	1.12
TS3	56.2/43.8	5.1	52.6/47.4	4.6	1.11
TS4	54.9/45.1	4.1	52.6/47.4	4.2	1.09
pTHF		9.9 ± 3.3		9.8	1.13

a * indicates the uncertain fit of the Lorentz distribution for the signals corresponding to the hard- and soft-block neighbors.

ML-derived NABLs align with those determined through NMR, the ML approach provides additional information on the shape of the BLDs. All BLDs showed a similar low dispersity of the BLD as shown in eq 3.

3 $${\mathrm{D̵}}_{BL}=\frac{weight‐average\;block\;length}{NABL}$$

4 $$NABL=\frac{\sum N_{i}M_{i}}{\sum N_{i}}$$

5 $$Weight‐average\;block\;length\;\left(WABL\right)=\frac{\sum N_{i}M_{i}^{2}}{\sum N_{i}M_{i}}$$

where *N*_*i*_ represents the abundance of chains with block length *i*, and *M*_*i*_ denotes the corresponding molecular weight of those chains.

A limitation of the current algorithm is its requirement for prior knowledge of the expected distribution. While the Schulz-Zimm distribution offers flexibility in shape, it is worth exploring automatic methods to determine which distribution is most suitable (i.e., provides the best fit), as it may not always be known in advance which distribution is appropriate.

## 4 Conclusions

In this study, BLDs were successfully determined from MS/MS data for a series of polyamide samples and five TPU samples, which a of yet not possible with alternative techniques such as NMR. To accommodate the diverse nature of copolymers, the previously developed algorithm^31^ was enhanced by incorporating preferences in bond cleavage based on bond type. Additionally, the workflow was adapted to accommodate various copolymer distributions by employing the Schulz-Zimm distribution, capable of processing distributions ranging from Flory to Gaussian. As validation, the polyamide BLDs showed good alignment from those obtained with the MC-based algorithm. By using five TPU samples with the same composition and pTHF segments, the hard block BLDs could be determined without any input such as the composition or fragmentation preference besides the fragment table. Validation of the BLD determination was conducted by comparing the NABL obtained from NMR spectroscopy with the values derived from our workflow. The difference between the NMR-based NABL and our workflow was on average 12% and a maximum of 22%, demonstrating consistency in the trend of hard-block lengths among the different TPU samples. This comparison serves as the current best option for validation, given the absence of standards with known BLDs and the absence of alternative methods to estimate the BLDs. In addition to the strong alignment of the new ML approach with the NMR NABL values, the determination of the BLD provides more detailed information on the chemistry of the TPU.

The determined BLDs were obtained under the assumption that these distributions are consistent across the molecular weight range. However, if this assumption does not hold, a bias may be introduced, as lower molecular weight species generally exhibit higher ionization efficiencies and may therefore be overrepresented in the fragment data. A limitation of the proposed method is the requirement for prior knowledge of the expected distribution type. Although the Schulz-Zimm distribution accommodates a wide range of profiles, it may not adequately describe systems with specifically engineered architectures or multimodal BLDs. In future work, it would be valuable to implement an approach in which each block length is treated as an independent variable, allowing for a distribution-free fitting process. However, this would introduce significant computational demands and substantially increase the uncertainty of the fit given the need to estimate the abundance of each block length, in our case 40 parameters, compared to the six used in the current approach. Addressing this complexity remains a key challenge for further development.

## Supplementary Material





## Notes

MS instrument settings, Exemplary mass spectra, LC separation to remove cyclic oligomers, ^13^C NMR deconvolution for PU samples, and 1H NMR Data (PDF)
Analytical solution workflow code: MATLAB code used for SWAMP (PDF)
