# Supplementary material for: Determination of Copolymer Block-Length Distributions Using Fragmentation Data Obtained from Tandem Mass Spectrometry
Source: Macromolecules. 2025 Jun 16;58(13):6430–9. doi: 10.1021/acs.macromol.5c00297 (PMC12257583; doi:10.1021/acs.macromol.5c00297)
Supplement: Supplementary file 1 [file ma5c00297_si_001.pdf]

Supporting Information

Determination of copolymer block-length distributions using fragmentation data obtained from tandem mass spectrometry

Tijmen S. Bos[1, 2,*], Rick S. van den Hurk[1, 2,*], Ynze Mengerink[3, 4], Ton Brooijmans[5], Ron A.H. Peters[1, 2, 5], Arian C. van Asten[1, 2, 6], Bob W.J. Pirok[1, 2]

[1] *Analytical Chemistry Group, Van 't Hoff Institute for Molecular Sciences, University of Amsterdam, 1098 XH, The Netherlands*

[2] *Centre for Analytical Sciences Amsterdam (CASA), Amsterdam, 1098 XH, the Netherlands*

[3] *Biomedical, DSM, Geleen, 6160 BB, the Netherlands*

[4] *Brightlands, Geleen, 6167 RD, the Netherlands*

[5] *Group Innovation & Sustainability, Testing, Analytics and Physics group, Covestro (Netherlands) B.V., Waalwijk, 5145 PE, the Netherlands*

[6] *Co van Ledden Hulsebosch Center (CLHC), Netherlands Center for Forensic Science and Medicine, Amsterdam, 1098 XH, the Netherlands*

* Equal contributions

corresponding author

Tijmen S. Bos, t.s.bos@uva.nl

Table of Contents

S-1 MS instrumental settings 3

S-2 Exemplary mass spectra 3

S-3 LC separation to remove cyclic oligomers 5

S-4 NMR deconvolutions 7

S-5 1H-NMR Data 8

S-1 MS instrumental settings

Table S-1 summarizes the instrument settings use for the mass spectrometer for the fragmentation of the polyurethane samples.

Table S-1: Overview of MS settings

Parameter	Setting
Polarity	Positive (+)
Collision Energy (V)	25
Gas Temp. (°C)	200
Gas Flow (L/min)	14
Sheat Gas Temp (°C)	350
Sheat Gas Flow (L/min)	11
m/z range	65 - 3200
Scan Rate (Hz)	4
VCap (V)	3500
Nozzle Voltage (V)	1000
Fragmentor (V)	380
Skimmer (V)	65
Octopole RF Peak (V)	750

S-2 Exemplary mass spectra

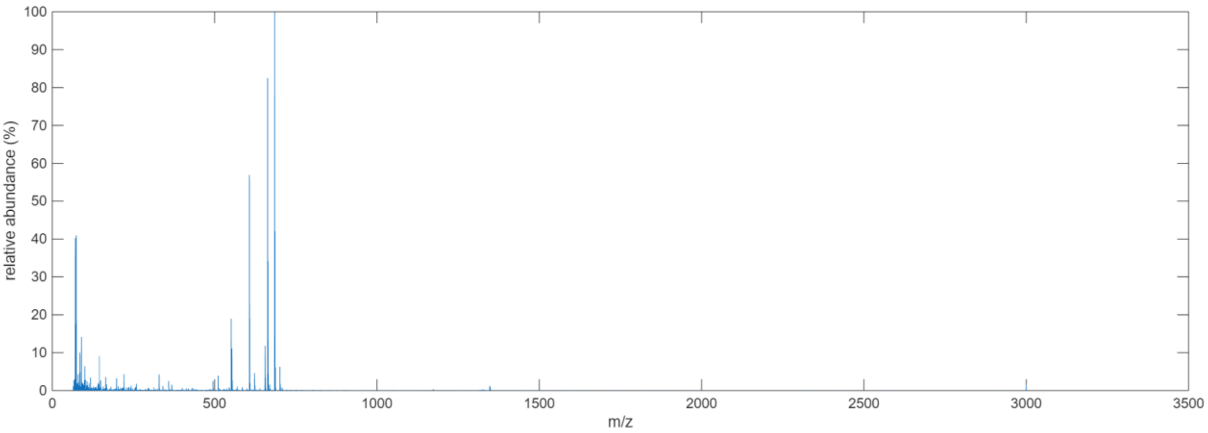

Figure S-1: Extracted mass spectrum of polyurethane sample TS2.

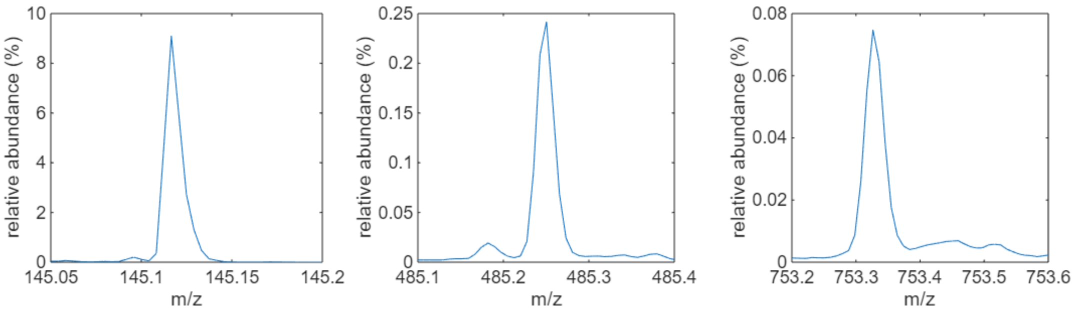

Figure S-2: Zoomed in mass spectra of polyurethane sample TS2 for signals corresponding to 2x THF (*m/z* = 145.12), 2x THF + 1x MDI-BDO (*m/z* = 485.26), and 1x THF + 2x MDI-BDO (*m/z* = 753.35) respectively.

S-3 LC separation to remove cyclic oligomers

While acetonitrile or methanol are typically used as organic modifiers in RPLC separations, most polymers dissolve poorly in them. Therefore, THF was used as an organic modifier in this study, which is one of the most common solvents used for separating polymers. The MDI-BDO oligomers are separated up to roughly n = 8. At that point, larger ones seem to co-elute as the polarity does not significantly change with additional repeat units. Besides the linear oligomers, some amine-terminated species as well as cyclic species were separated and identified. More importantly, all of these lower molecular weight analytes were eluted well before 30 minutes. The polyurethane (PU) product itself eluted around the 30-minute mark, showing that it was separated from possible oligomeric impurities (Fig. S-1). To minimize impurities in the fragment table, only signals from the elution time range of the polyurethane (29-33 minutes) were considered, excluding signals from any impurities that eluted earlier. The corresponding fragment table is displayed in Fig. S-2).

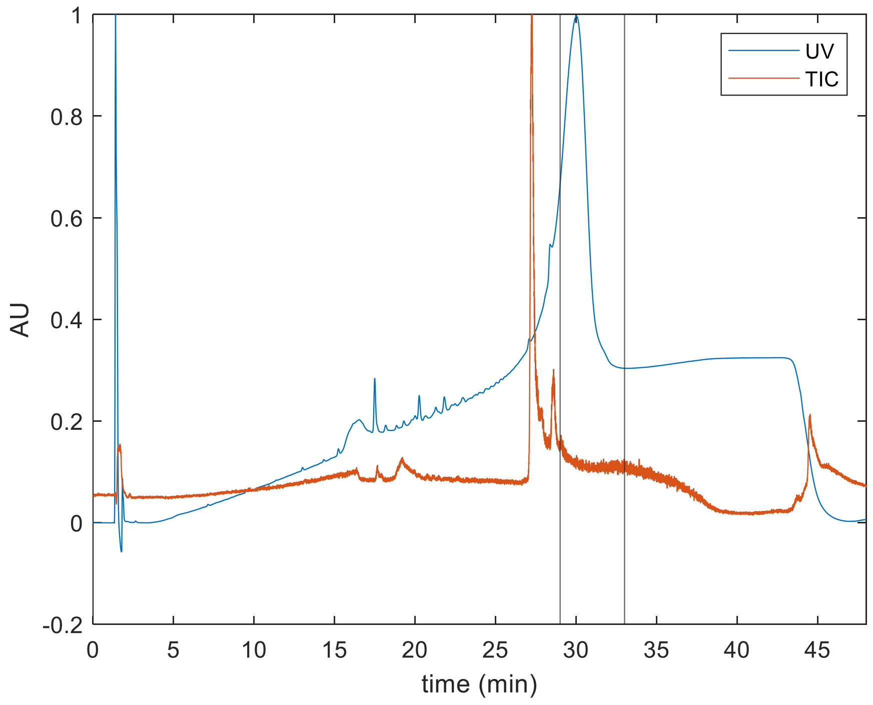

Figure S-3: TIC and RPLC-UV (254 nm) chromatogram of PU sample 2 shot 4. Vertical lines indicated data used to form the fragment tables.

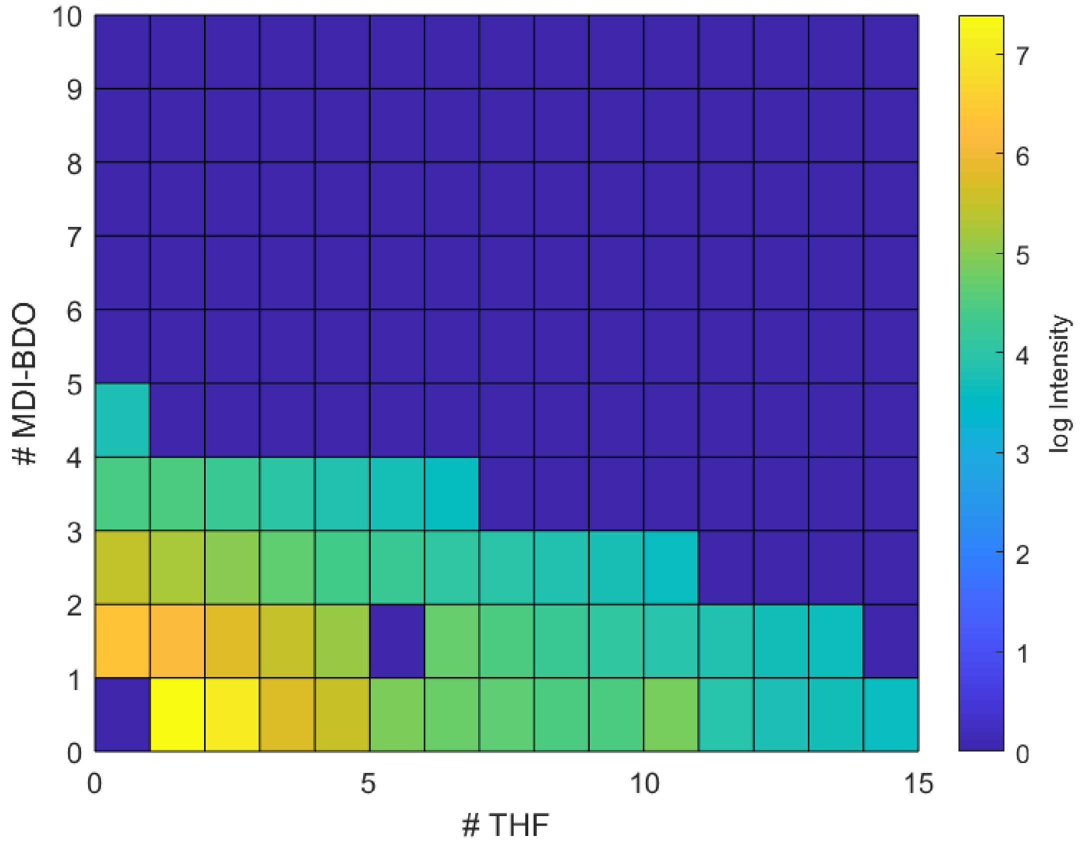

Figure S-4: Fragment table obtained at 25V of PU sample 2 shot 4 in the timeframe that was indicated with vertical lines in Fig. S-1. Note that the color-scale is logarithmic to better visualize low abundant fragments and that THF_5-MDI-BDO_1 fragment was not observed.

S-4 NMR deconvolutions

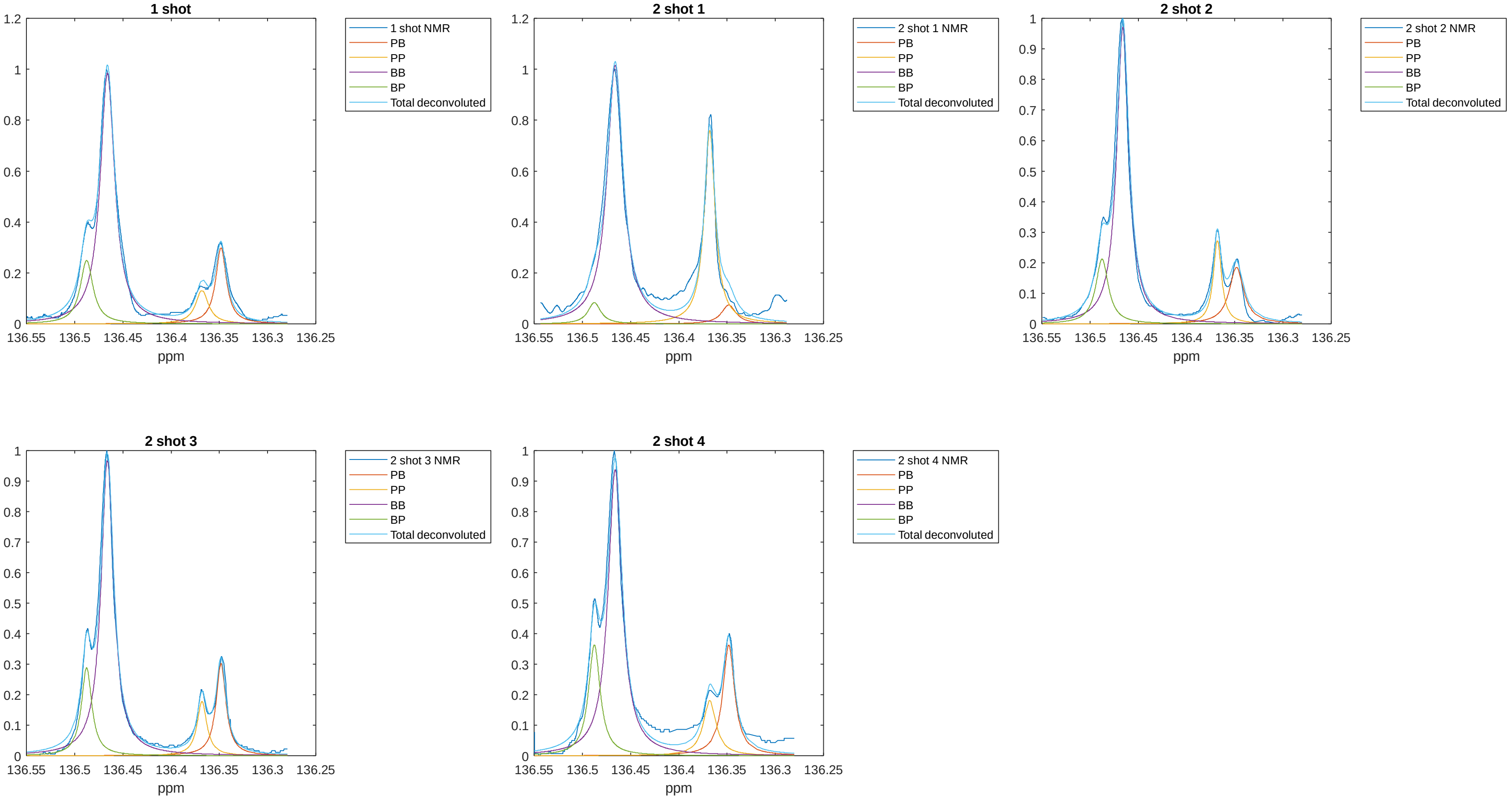

Figure S-5: NMR spectra of the 5 PU samples. Dark blue traces indicate the raw data and the underlying colored lines are the deconvoluted PB (Soft-Hard block, orange), PP (Soft-Soft block, yellow), BB (Hard-Hard block, purple), and BP (Hard-Soft block, green). The total contribution of the deconvoluted NMR spectra is shown in light blue.

S-5 1H-NMR Data

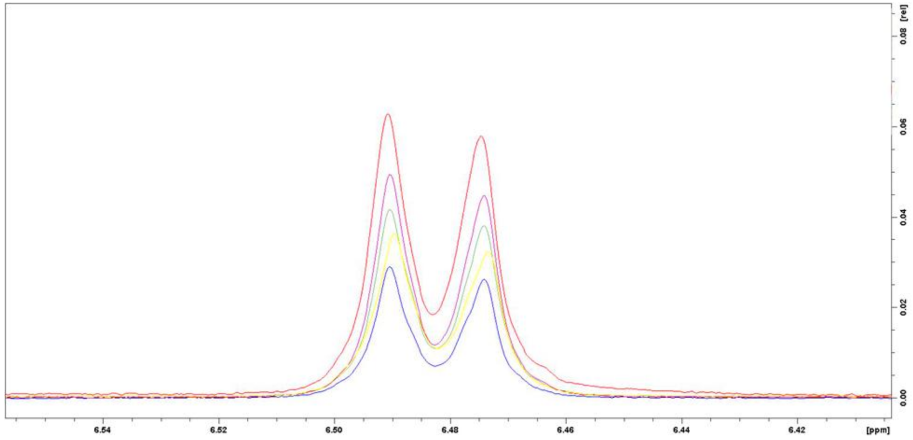

Figure S-6: Overlay plot of the MDA-peak of sample one-shot (blue), two-shot-1 (red), -2 (green), -3 (purple) and two-shot-4 (yellow)

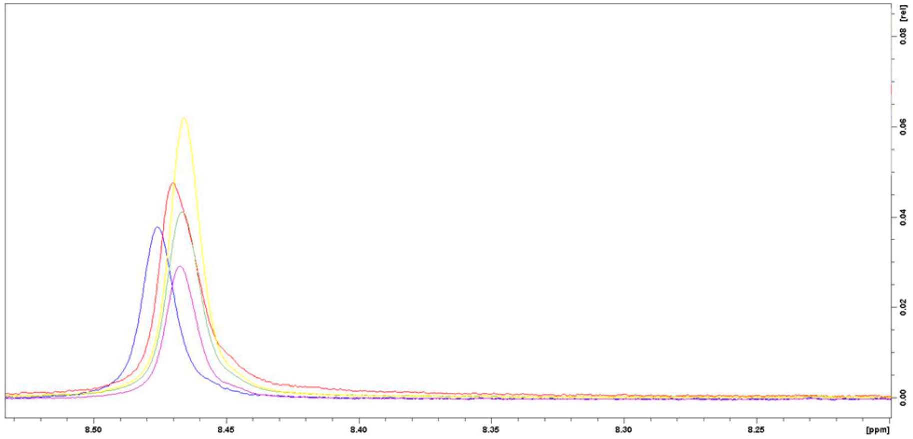

Figure S-7: Overlay plot of the Amide-peak of sample one-shot (blue), two-shot-1 (red), -2 (green), -3 (purple) and two-shot-4 (yellow)
