# Supplementary material for: Determination of Copolymer Block-Length Distributions Using Fragmentation Data Obtained from Tandem Mass Spectrometry
Source: Macromolecules. 2025 Jun 16;58(13):6430–9. doi: 10.1021/acs.macromol.5c00297 (PMC12257583; doi:10.1021/acs.macromol.5c00297)
Supplement: Supplementary file 2 [file ma5c00297_si_002.pdf]

Supporting Information – Analytical solution workflow code

Determination of copolymer block-length distributions using fragmentation data obtained from tandem mass spectrometry

Tijmen S. Bos[1, 2,*], Rick S. van den Hurk[1, 2,*], Ynze Mengerink[3, 4], Ton Brooijmans[5], Ron A.H. Peters[1, 2, 5], Arian C. van Asten[1, 2, 6], Bob W.J. Pirok[1, 2]

[1] *Analytical Chemistry Group, Van 't Hoff Institute for Molecular Sciences, University of Amsterdam, 1098 XH, The Netherlands*

[2] *Centre for Analytical Sciences Amsterdam (CASA), Amsterdam, 1098 XH, the Netherlands*

[3] *Biomedical, DSM, Geleen, 6160 BB, the Netherlands*

[4] *Brightlands, Geleen, 6167 RD, the Netherlands*

[5] *Group Innovation & Sustainability, Testing, Analytics and Physics group, Covestro (Netherlands) B.V., Waalwijk, 5145 PE, the Netherlands*

[6] *Co van Ledden Hulsebosch Center (CLHC), Netherlands Center for Forensic Science and Medicine, Amsterdam, 1098 XH, the Netherlands*

* Equal contributions

corresponding author

Tijmen S. Bos, t.s.bos@uva.nl

MATLAB 2024a code for determining the block length distribution of a copolymer from a fragment table obtained from MS/MS data.

```
function [BLDA,BLDB] =
BlocklengthdeterminatorMSMS(fragmentmatrix,nmax,maxfragsize,x0)
%  MATLAB 2024a code for determining the block length distribution of a copolymer
from a fragment table obtained with MS/MS.
%
% Input:    fragmentmatrix: fragment matrix. [A0B0 A1B0 A2B0; A1B0 A2B1 A2B2; A2B0
A2B1 A2B2]
%           nmax: Maximum block length that is evaluated.
%           maxfragsize: Maximum fragment size to be evaluated. Not advides
%           to go above 7 as the computation time increases drastically.
%           x0: Starting point of optimization. [mu BLD A, sigma BLD A, mu BLD B,
sigma BLD B, free monomer A]
%
% Output:   BLDA: block length distribution of A
%           BLDB: block length distribution of B
%
% This function is part of a publication by Bos, van den Hurk, Mengerink,
% Brooijmans, Peters, van Astern, and Pirok.
% entitled “Determination of copolymer block-length distributions using
fragmentation data obtained from tandem mass spectrometry”.
% 2024, Universiteit van Amsterdam.
%
%
% For inquaries on the algorithmn contact Tijmen S. Bos at t.s.bos@uva.nl


%% resizing fragment table
maxmaxfragsize = max(maxfragsize);

if size(fragmentmatrix,1) ~= size(fragmentmatrix,2)
    fragmentmatrixpre = zeros(maxmaxfragsize+1);
    fragmentmatrixpre(1:min([size(fragmentmatrix,1)
maxmaxfragsize+1]),1:min([size(fragmentmatrix,2) maxmaxfragsize+1]))  =
fragmentmatrix(1:min([size(fragmentmatrix,1)
maxmaxfragsize+1]),1:min([size(fragmentmatrix,2) maxmaxfragsize+1]));

    fragmentmatrix = fragmentmatrixpre;
end

if numel(maxfragsize) == 2
    fragmentmatrixtemp = zeros(size(fragmentmatrix));
    fragmentmatrixtemp(1:maxfragsize(1)+1,1:maxfragsize(2)+1) =
fragmentmatrix(1:maxfragsize(1)+1,1:maxfragsize(2)+1);
    fragmentmatrix = fragmentmatrixtemp;
end



%% normalize fragment table
for i = 2:maxmaxfragsize+1
    sel = 1:i;
    temp = [];
    for k = 1:numel(sel)
        try
            temp(k) = fragmentmatrix(sel(k),sel(end-k+1));
```

```
        catch
        end
    end

    temp = temp./(sum(temp));

    for k = 1:numel(sel)
        try
            fragmatnorm(sel(k),sel(end-k+1))  = temp(k);
        catch
        end
    end
end


fragmatnorm(isnan(fragmatnorm)) = 0;
fragmatnorm(fragmatnorm == 1) = 0;


%% Machine learning settings
options = optimoptions('lsqnonlin',...
    'Algorithm','trust-region-reflective',...
    'MaxIterations',inf,...
    'MaxFunctionEvaluations',inf,...
    'OptimalityTolerance',0,...
    'MaxPCGIter',1000,...
    'TolPCG',1e-6,...
    'FunctionTolerance',1e-12,...
    'StepTolerance',1e-6,...
    'UseParallel',true,...
    'PlotFcn',optimplotxcustom,...
    'Display','iter-detailed');

Aeq = [];
Beq = [];
A = [];
b = [];


lb = [0 1 0 1 0 0 0];
ub = [inf maxsize inf maxsize 1 1 1];

fun = @(P) supfun(P, nmax-1,maxmaxfragsize,fragmatnorm);

%% Start machine learning process
Popt = lsqnonlin(fun,x0,lb,ub,A,b,Aeq,Beq,[],options);

%% Restructure out
x= 1:nmax;
BLDA = ((Popt(1)^Popt(1)).*exp(-Popt(1).*x./Popt(2)).*(x.^(Popt(1)-
1)))./(gamma(Popt(1)).*(Popt(2).^Popt(1))) ;
BLDB = ((Popt(3)^Popt(3)).*exp(-Popt(3).*x./Popt(4)).*(x.^(Popt(3)-
1)))./(gamma(Popt(3)).*(Popt(4).^Popt(3))) ;

BLDA = BLDA./(sum(BLDA));
BLDB = BLDB./(sum(BLDB));
end

%% Local support functions
```

```
%% Function to alter matrix such only relvant data points are included
function [supfunres] = supfun(P, nmax,maxfragsize,fragmatnorm)
[tempMS] = probability_mat_copoly(P, nmax,maxfragsize,fragmatnorm) ;
tempMS = tempMS(:);

for i = 1:max(size(fragmatnorm))+1
    sel = 1:i;
    temp = [];
    for k = 1:numel(sel)
        try
            temp(k) = fragmatnorm(sel(k),sel(end-k+1));
        catch
        end
    end

    temp = temp./(sum(temp));

    for k = 1:numel(sel)
        try
            fragmatnorm2(sel(k),sel(end-k+1))  = temp(k);
        catch
        end
    end
end

fragmatnormtemp = fragmatnorm2(:);
supfuntempMSres =  [tempMS(fragmatnorm2(:) > 0)] -
[fragmatnormtemp(fragmatnormtemp(:) > 0)];
supfunres = supfuntempMSres(:);

end

%% Function that prepares the input and output of the analytical fragment
generation
function [probmat] = probability_mat_copoly(P, nmax,maxfragsize,fragmatnorm)
%(nmax,  pA, pB)

x = 1:nmax;
pA = ((P(1)^P(1)).*exp(-P(1).*x./P(2)).*(x.^(P(1)-
1)))./(gamma(P(1)).*(P(2).^P(1))) ;
pB = ((P(3)^P(3)).*exp(-P(3).*x./P(4)).*(x.^(P(3)-
1)))./(gamma(P(3)).*(P(4).^P(3))) ;

pA = pA./(sum(pA));
pB = pB./(sum(pB));

probmat = fragmentgen_opt_bond(maxfragsize,pA,pB,P(5:7));
probmat = probmat .* logical(fragmatnorm);

for i = 1:maxfragsize+1
    sel = 1:i;
    temp = [];
    for k = 1:numel(sel)
        try
            temp(k) = probmat(sel(k),sel(end-k+1));
        catch
        end
    end
```

```
    temp = temp./(sum(temp));

    for k = 1:numel(sel)
        try
            probmat(sel(k),sel(end-k+1))  = temp(k);
        catch
        end
    end
end

probmat(isnan(probmat)) = 0;

end


%% Algortimn of the analytical fragment table generation
function [probmat] = fragmentgen_opt_bond(maxlength,pA,pB,bonstrratio)
probmat = zeros(maxlength+1);
meanpA = sum(pA.*(1:numel(pA)));
meanpB = sum(pB.*(1:numel(pB)));
meandoubleblock = meanpA+meanpB;

bonstrratio(4) = bonstrratio(2);
%%AA (11;0) bonstrratio(1)
%%AB (10;-1) bonstrratio(2)
%%BB (00;0) bonstrratio(3)
%%BA (01;;1) bonstrratio(4)


BLDmat = pA'*pB;
for n = 1:maxlength
    for i = 1:numel(pA)
        for j = 1:numel(pB)
            if BLDmat(i,j) > 10^-6
                blockcomb = i+j;
                if n > 1
                    sequence = [ones([1 i]) zeros([1 j])];

                    probmattemp =
largern(sequence,maxlength,BLDmat,n,0,blockcomb,bonstrratio);
                    probmat =
probmat+probmattemp.*BLDmat(i,j).*((i+j)/meandoubleblock);
                else

                    % Include fragmentation preference
                    sequence = [ones([1 i]) zeros([1 j])];
                    secondcleave = (diff([sequence 1]));

                    secondcleave(secondcleave == 1) = 3;
                    secondcleave(secondcleave == -1) = 1;

                    firstcleave = (diff([0 sequence]));
                    firstcleave(firstcleave == 1) = 3;
                    firstcleave(firstcleave == -1) = 1;


                    for g = 1:numel(firstcleave)
                        if firstcleave(g) == 0
                            if sequence(g) == 0
```

```
                                firstcleave(g) = 2;
                            end
                        end

                        if secondcleave(g) == 0
                            if sequence(g+1) == 0
                                secondcleave(g) = 2;
                            end
                        end
                    end

                    bondstrf =
bonstrratio(firstcleave+1).*bonstrratio(secondcleave+1);
                    counter(2) = sum(sequence .* bondstrf);
                    counter(1) = sum(~sequence .* bondstrf);
                    counter =
counter./(sum(counter)).*BLDmat(i,j).*((i+j)/meandoubleblock);
                    % Add probabilitie to fragment matrix
                    for f = 1:numel(counter)
                        probmat(1+n-(f-1),1+(f-1)) = probmat(1+n-(f-1),1+(f-1)) +
counter(f);
                    end
                end
            end
        end
    end
end
end

%% Sub function for Algortimn of the analytical fragment table generation
function [probmat] =
largern(sequence,maxlength,BLDmat,n,blockcombor,blockcomboror,bonstrratio)
probmat = zeros(maxlength+1);

for i = 1:size(BLDmat,1)
    for j = 1:size(BLDmat,2)
        if BLDmat(i,j) > 10^-6
            sequencenew = [[ones([1 i]) zeros([1 j])] sequence];
            if n > blockcombor+i+j+1

                probmattemp =
largern(sequencenew,maxlength,BLDmat,n,blockcombor+i+j,blockcomboror,bonstrratio);
                probmat = probmat+probmattemp.*BLDmat(i,j);
            else

                relsel = sequencenew(blockcombor+i+j+1-(n-1):end);
                numbA = cumsum(relsel);
                numbA = [numbA(n) numbA(n+1:end)-(numbA(1:end-n))];


                secondcleave = abs(diff([sequencenew(end-(blockcomboror-1):end)
1]));

                if blockcombor+i+j+1-(n-1)-1 == 0
                    firstcleave = abs(diff([0 sequencenew(blockcombor+i+j+1-(n-
1):end-n+1)]));
                else
                    firstcleave = abs(diff([sequencenew(blockcombor+i+j+1-(n-1)-
1:end-n+1)]));
```

```
                end


                secondcleave(secondcleave == 1) = 3;
                secondcleave(secondcleave == -1) = 1;

                firstcleave(firstcleave == 1) = 3;
                firstcleave(firstcleave == -1) = 1;


                for g = 1:numel(firstcleave)
                    if firstcleave(g) == 0
                        if sequence(g) == 0
                            firstcleave(g) = 2;
                        end
                    end

                    if secondcleave(g) == 0
                        if sequence(g+1) == 0
                            secondcleave(g) = 2;
                        end
                    end
                end


                bondstrf = 
bonstrratio(firstcleave+1).*bonstrratio(secondcleave+1);

                counter = sum(((0:n) == numbA').*bondstrf');
                counter = counter./(sum(counter)).*BLDmat(i,j);

                for f = 1:numel(counter)
                    probmat(1+n-(f-1),1+(f-1)) = probmat(1+n-(f-1),1+(f-1)) + 
counter(f);
                end

            end
        end
    end
end

end

%% Custom plot function to follow the properties of the BLD over the itterations
function [stop] = optimplotxcustom(x,optimValues,state,varargin)
stop = false;
switch state
    case 'iter'
        % Reshape if x is a matrix
        x = x(:);
        xLength = length(x);


        if optimValues.iteration == 0
            plotx = bar(x);
            
title(getString(message('MATLAB:optimfun:funfun:optimplots:TitleCurrentPoint')),'i
nterp','none');
```

```
ylabel(getString(message('MATLAB:optimfun:funfun:optimplots:LabelCurrentPoint')),'
interp','none');
            set(plotx,'edgecolor','none')
            set(gca,'xlim',[0,1 + xLength],'ylim',[0 inf])
            set(plotx,'Tag','optimplotx');

        else
            plotx = findobj(get(gca,'Children'),'Tag','optimplotx');
            set(plotx,'Ydata',x);
        end

end
end
```
